# Supplementary material for: Gene Therapy Using Efficient Direct Lineage Reprogramming Technology for Neurological Diseases
Source: Nanomaterials (Basel). 2023 May 19;13(10):1680. doi: 10.3390/nano13101680 (PMC10222404; doi:10.3390/nano13101680)
Supplement: Supplementary file 1 [file nanomaterials-13-01680-s001.zip › nanomaterials-2389412-supplementary.pdf]

Supporting Information

Gene Therapy Using Efficient Direct Lineage Reprogramming Technology for Neurological Diseases

Yujung Chang [1,2,†], Sungwoo Lee [3,†], Jieun Kim [4], Chunggoo Kim [1], Hyun Soo Shim [1], Seung Eun Lee [5], Hyeok Ju Park [6], Jeongwon Kim [3], Soohyun Lee [3], Yong Kyu Lee [6], Sungho Park [3,*] and Junsang Yoo [1,*]

[1] Laboratory of Regenerative Medicine for Neurodegenerative Disease, Stand Up Therapeutics, Hannamdaero 98, Seoul 04418, Republic of Korea; drchang@stutps.com (Y.C.)
[2] Department of Molecular Biology, Nuturn Science, Sinsadong 559-8, Seoul 06037, Republic of Korea
[3] Department of Chemistry, Sungkyunkwan University, 2066, Seobu-ro, Jangan-gu, Suwon-si 16419, Republic of Korea; sungwoo22@skku.edu (S.L.); kjw1029@skku.edu (J.K.); s11hy1n@skku.edu (S.L.)
[4] Department of Bio-Health Technology, College of Biomedical Science, Kangwon National University, 1 Kangwondeahak-gil, Chuncheon 24341, Republic of Korea; jieunkim@kangwon.ac.kr
[5] Research Animal Resource Center, Korea Institute of Science and Technology, Hwarang-ro 14-gil, Seongbuk-gu, Seoul 02792, Republic of Korea; selee@kist.re.kr (S.E.L.);
[6] Database Laboratory, Department of Computer Science and Engineering, Dongguk University-Seoul, Pildong-ro 1-gil 30, Jung-gu, Seoul 04620, Republic of Korea; phj1987@gmail.com (H.Y.P.); yklee@dongguk.edu (Y.K.L.)
* Correspondence: spark72@skku.edu (S.P.); dryoo0912@gmail.com (J.Y.)
† These authors contributed equally to this work.

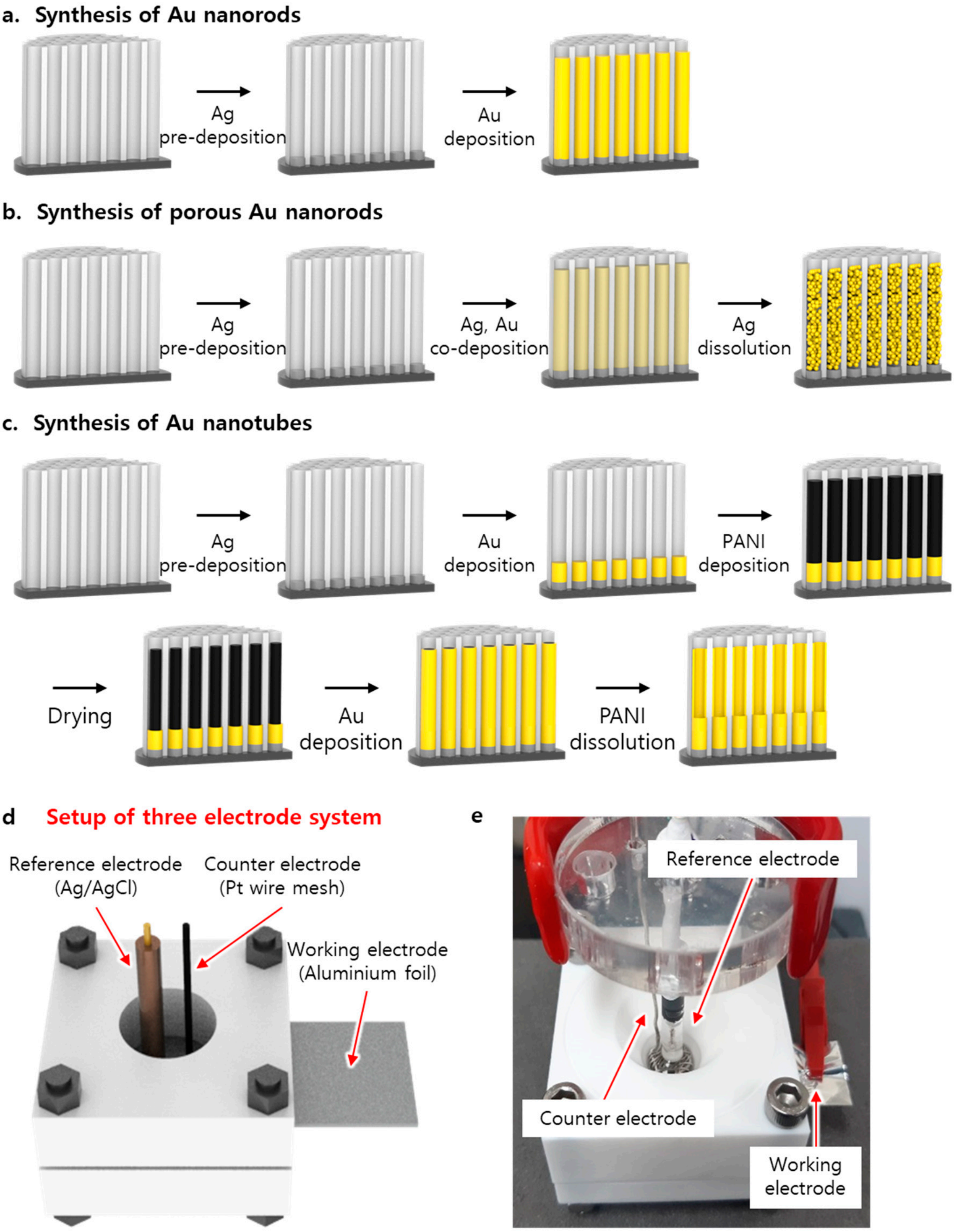

Figure S1. Schematic illustration on synthesis of nanorods and three-electrode system for the electrochemical deposition method. Schematic illustration representing synthetic procedure of (a) Au nanorods, (b) porous Au nanorods, (c) Au nanotubes, and (d) setup of three- electrode system for the electrochemical deposition method. (e) Photograph of three-electrode system consisting of reference electrode, counter electrode, and working electrode. Adapted from Lee et al. 2022, Acta Biomaterialia [29]. Copyright from {2022}Acta Biomaterialia.
